# Temporal Trends in Stroke Prevalence and Associated Risk Factors in the United States: Insights From National Health and Nutrition Examination Survey (NHANES) 1999-2020

**DOI:** 10.7759/cureus.103677

**Published:** 2026-02-15

**Authors:** Arjun Mahesh, Anjali Rajpoot

**Affiliations:** 1 Neuroscience/Public Health Research, Henrico High School, Richmond, USA; 2 Biology, Banasthali Vidyapith, Jaipur, IND

**Keywords:** diabetes, hypertension, nhanes, obesity, prevalence, stroke, united states

## Abstract

Introduction: Stroke is a leading cause of mortality and disability in the United States (US). Although hypertension, diabetes, and obesity are considered traditional risk factors for stroke, the extent to which these factors have changed their relationship and the prevalence of stroke has not been well-characterized since 1999. Therefore, the objective of this study is to investigate the temporal trends in stroke prevalence among US adults and to determine how stroke risk factors have changed over the past 20 years.

Methods: To achieve this objective, we conducted a secondary analysis of NHANES 1999-2020 data using a cross-sectional approach. Further, the prevalence of stroke was self-reported physician diagnosis, and major risk factors (hypertension, diabetes, obesity, smoking, and hyperlipidemia) were measured on standardized NHANES definitions. Temporal trends and associations within cycles were assessed using the survey-weighted logistic regression and age-adjusted prevalence estimates.

Results: The findings indicate that the age-adjusted prevalence of stroke among US adults aged ≥20 years increased significantly across all NHANES cycles (1999-2020) during the study period. The highest burden was observed in older adults, men, and non-Hispanic Black persons. Hypertension and diabetes were the strongest risk factors, while the contribution of obesity increased, and that of smoking decreased.

Conclusion: The study concluded that stroke prevalence in the US has increased in the last 20 years due to the continuous presence of vascular risk factors and the alteration of lifestyle patterns. Such findings highlight the importance of preventive and risk-mitigation interventions, especially for high-risk populations.

## Introduction

Stroke is one of the most significant causes of mortality and long-term disability in the United States (US), and it is also among the most significant health burdens worldwide [1,2]. Approximately 800,000 Americans have a stroke each year, and about one in 20 deaths is related to stroke annually [3]. In addition to mortality, survivors frequently experience chronic neurological impairment that restricts the ability to live independently, increases long-term care needs, and adds to healthcare spending [4]. Stroke prevention and risk factor control are at the top of the clinical and public health agenda, as the absolute number of stroke cases in the US population is expected to rise with an aging population. Although acute management and secondary prevention have made modest gains in reducing case fatality, the prevalence of stroke at the population level continues to rise due to demographic aging and long-term exposure to modifiable vascular risks [5].

One major issue associated with the reduction of stroke burden is that risk factors and prevalence are not evenly distributed among demographic groups. Previous national surveys have consistently demonstrated differences in stroke incidence and outcomes based on age, sex, race/ethnicity, and socioeconomic status [6,7]. For example, non-Hispanic Black adults show the highest prevalence and mortality rates of stroke, a trend largely attributed to the higher prevalence of hypertension and diabetes, compounded by structural barriers to healthcare access [8]. Hispanic and Native American adults are also at elevated risk, though their patterns differ in comparison to non-Hispanic Whites [9]. Gender variations also exist, with men experiencing a higher incidence of stroke at younger ages, while women face a higher lifetime risk due to longevity and unique exposures such as pregnancy-related hypertensive disorders [10]. These trends indicate that stroke is not only a medical problem but also a measure of social inequity, underscoring the need for subgroup analysis to examine how trends are distributed over time.

The evolution of modifiable vascular risk factors is another important determinant shaping the stroke landscape. Hypertension remains the most prevalent risk factor for both incident and recurrent strokes, a role confirmed by pooled international research [11]. Diabetes mellitus has become a significant contributor, with prevalence rates in the U.S. nearly doubling since the early 2000s [12]. At the same time, the obesity crisis has radically transformed the cardiovascular and cerebrovascular risk profile of the U.S., particularly among younger adults now entering middle age [13]. Conversely, smoking prevalence has dropped substantially because of decades of public health promotion, taxation, and regulation [14]. Hyperlipidemia, while once a stronger predictor of stroke, now appears confounded by other factors; widespread use of statins has reduced its independent effect, raising the possibility that its role is mediated by stronger comorbidities such as hypertension and diabetes [15]. Together, these shifts underscore the importance of reevaluating which risk factors remain most relevant to stroke prevention programs today.

The National Health and Nutrition Examination Survey (NHANES) provides a unique platform to assess these questions [16]. NHANES is conducted in two-year cycles and includes structured household interviews, clinical examinations, and laboratory measurements, making it one of the most reliable and comprehensive databases for monitoring chronic disease trends [16]. Unlike hospital-based cohorts, NHANES offers population-level estimates representing individuals across demographic, socioeconomic, and geographic strata of the civilian U.S. population [16]. This design makes it possible not only to estimate the overall prevalence of stroke but also to perform subgroup analyses by race/ethnicity, sex, age, and education, which are critical for identifying inequities. NHANES has previously been used to assess trends in cardiovascular and metabolic risk factors [17,18], but far fewer studies have examined stroke prevalence over two decades alongside parallel changes in vascular risk factors.

Thus, this study aimed to examine temporal changes in the prevalence of stroke and its key risk factors among US adults using NHANES data from 1999 to 2020. Specifically, our objectives were 1) to determine how stroke prevalence changed over 20 years, 2) to evaluate subgroup differences by age, sex, and race/ethnicity, and 3) to analyze the evolving role of major vascular risk factors such as hypertension, diabetes, obesity, smoking, and hyperlipidemia. Changes were assessed as temporal trends in prevalence across survey cycles, and the evolving role of risk factors was evaluated through comparisons of adjusted associations with stroke across NHANES cycles rather than formal interaction testing. By leveraging nationally representative data, this study presents an updated portrait of the epidemiology of stroke in the US. These findings are critical for informing clinical practice, guiding public health policy, and addressing persistent disparities in cerebrovascular health.

## Materials and methods

Study data and data sources

This was a cross-sectional secondary analysis of the NHANES 1999-2020 data. The National Center for Health Statistics (NCHS) conducts a nationally representative survey of the noninstitutionalized civilian population in the US, called NHANES, which is conducted in two-year cycles. The survey is conducted in a multistage stratified probability sample design and consists of structured household interviews, as well as standardized physical examinations and laboratory tests. Since all the data are publicly available, anonymized, and deidentified, this study was not required to undergo the institutional review board (IRB) approval.

Study population

The analytic sample consisted of adults aged ≥20 years who participated in NHANES between 1999 and 2020. Stroke status was determined based on responses to the question: “Has a doctor or health professional ever told you that you had a stroke?” Participants who responded “don’t know” refused to answer, or had missing responses to the stroke question were excluded from the analysis. Similar exclusions were applied to other covariates with missing or refusal responses prior to listwise deletion. Adults aged ≥20 years who provided a valid response to the stroke question were included, yielding ~55,000 participants across all NHANES cycles from 1999 to 2020. All analyses were done using NHANES survey weights, strata, and primary sampling units (PSUs) to come up with nationally representative estimates of the adult population in the US.

Measures

For this study, the stroke prevalence was based on the self-reported physician diagnosis and was coded as a binary variable (yes/no). Hypertension was defined as self-reported physician diagnosis of hypertension (NHANES questionnaire variable BPQ020) or measured systolic blood pressure ≥140 mmHg or diastolic blood pressure ≥90 mmHg based on the average of up to three standardized examination measurements (variables BPXSY and BPXDI). Sensitivity analyses were conducted using an alternative threshold of ≥130/80 mmHg, and results were consistent.

Diabetes mellitus was defined as self-reported physician diagnosis of diabetes (DIQ010) or a measured glycated hemoglobin level ≥6.5% (LBXGH). Obesity was categorized as body mass index (BMI) ≥30 kg/m², calculated from measured height and weight obtained during the physical examination (BMXBMI).

Smoking status was defined using the standard NHANES definition of current smoking, based on self-report of having smoked at least 100 cigarettes in one’s lifetime (SMQ020) and reporting current smoking “every day” or “some days” at the time of the interview (SMQ040).

Hyperlipidemia was defined as total serum cholesterol ≥240 mg/dL (LBXTC) or current use of lipid-lowering medications, ascertained from the NHANES prescription medication questionnaire (RXQ_RX), which was consistently available across all survey cycles.

Demographic covariates included age (modeled as both continuous and categorical), sex, race/ethnicity (non-Hispanic White, non-Hispanic Black, Hispanic, and other), and education level. Age-standardized prevalence estimates were calculated within each NHANES cycle using survey-weighted methods and standardized to the 2000 U.S. Census population, employing three age categories: 20-39, 40-59, and ≥60 years.

Statistical analysis

We initially estimated the weighted prevalence of stroke and each risk factor for each NHANES survey cycle. The 2000 US Census population was used as the standard to derive age-standardized prevalence. The survey cycle was modeled as a continuous variable to assess temporal trends using survey-weighted logistic regression, and linear trends were evaluated.

Subgroup analyses were stratified according to sex, race/ethnicity, and socioeconomic status (education level). Multivariable logistic regression models were fitted using pooled survey cycles to assess associations between stroke and individual risk factors, with adjustment for demographic covariates. Sensitivity analyses were conducted by sequential inclusion of covariates to assess the stability of associations.

All analyses accounted for the complex NHANES survey design. For pooled analyses across 1999-2020, Mobile Examination Center (MEC) examination weights (WTMEC2YR) were used, as stroke and key risk factors were derived from examination and laboratory components. For combined cycles, two-year MEC weights were divided by the total number of survey cycles included to create appropriate pooled weights, following NHANES analytic guidelines. This approach ensured equal contribution of each two-year cycle to the pooled national estimates. Survey strata (SDMVSTRA) and PSUs (SDMVPSU) were specified as provided by NHANES and were not redefined across cycles. Strata and PSU variables were treated as unique across cycles, consistent with NHANES pooled analysis recommendations.

For the partial 2019-2020 cycle, the NHANES provided a combined examination weight that was used as recommended by the NCHS and incorporated into the pooled weighting scheme to maintain national representativeness.

Variance estimation was performed using Taylor series linearization. All analyses were conducted using R version 4.3 (R Foundation for Statistical Computing, Vienna, Austria) with the survey package. Statistical significance was defined as a two-sided p value of <0.05. Missing data were handled using listwise deletion; overall missingness was minimal (<5% for most variables) and unlikely to meaningfully bias results.

Temporal trends were modeled by treating the NHANES survey cycle as a continuous variable, assuming linear change over time. Changes in the contribution of risk factors were assessed by comparing adjusted odds ratios (ORs) across survey cycles rather than by formal interaction testing between cycle and risk factors. No joinpoint or breakpoint analyses were conducted. Because analyses focused on a predefined set of major vascular risk factors, no formal correction for multiple testing was applied.

Ethics statement

The analysis was based on publicly available, deidentified NHANES data and therefore did not require IRB approval. NHANES survey protocols are reviewed and approved by the NCHS Research Ethics Review Board, and all participants provide informed consent before data collection.

## Results

Descriptive characteristics

In all NHANES cycles (1999-2020), an analytic sample comprised about 55,000 adults aged 20 years or older. The adjusted prevalence of self-reported physician-diagnosed stroke was 2.9% (95% confidence interval (CI): 2.6-3.2; n = 1,595). There was an age-related increase in stroke prevalence with age, which was 8.5% in adults aged ≥65 years and 1.2% in adults aged 20-44 years. The prevalence was lower in women than in men (3.2% (n = 852) vs. 2.7% (n = 743)). Non-Hispanic Black adults were the most affected by the prevalence of stroke (4.4%), followed by non-Hispanic White (2.8%), Hispanic (2.5%), and other/multiracial (2.3%). Additionally, the study population was generally exposed to major risk factors: hypertension (34.6%), obesity (31.8%), diabetes (9.9%), hyperlipidemia (16.5%), and current smoking (20.1%). Stroke survivors bore the greatest burden of these conditions, and more than three-quarters of stroke-positive respondents indicated having a history of hypertension, and almost half indicated having a history of diabetes.

Temporal trends

The age-standardized prevalence of stroke increased from 2.4% in 1999-2000 to 3.2% in 2019-2020 (p < 0.001). Hypertension prevalence rose through the mid-2000s, plateaued, and then showed a modest decline after 2010. Diabetes prevalence increased steadily, from 6.2% to 12.8% over the study period (p < 0.001). The prevalence of obesity nearly doubled, rising from 19.4% in 1999-2000 to 36.1% in 2019-2020 (p < 0.001). By contrast, smoking prevalence declined consistently, reaching 14.8% in 2019-2020 (p < 0.001). Changes in the strength of association between stroke and individual risk factors over time were inferred from comparisons of adjusted effect estimates across NHANES cycles. Table 1 presents selected NHANES cycles to illustrate long-term trends; all survey cycles from 1999 to 2020 were included in the analyses. Hyperlipidemia was analyzed in regression models, but is not shown in this table to improve readability.

**Table 1:** Age-standardized prevalence of stroke and major risk factors among U.S. adults, NHANES 1999-2020 Data are age-standardized to the 2000 US census population. NHANES sampling weights are applied. Percentages are weighted estimates NHANES: National Health and Nutrition Examination Survey

NHANES cycle	Stroke (%)	Hypertension (%)	Diabetes (%)	Obesity (%)	Smoking (%)
1999-2000	2.4 (n = 218)	32.8 (n = 2,988)	6.2 (n = 565)	19.4 (n = 1,767)	23.5 (n = 2,141)
2003-2004	2.7 (n = 266)	35.4 (n = 3,492)	7.8 (n = 769)	26.1 (n = 2,574)	22.0 (n = 2,169)
2007-2008	2.9 (n = 292)	34.9 (n = 3,516)	9.4 (n = 947)	20.5 (n = 2,064)	19.5 (n = 1,964)
2011-2012	3.0 (n = 321)	33.2 (n = 3,552)	11.1 (n = 1,187)	33.8 (n = 3,615)	16.9 (n = 1,807)
2015-2016	3.1 (n = 340)	32.7 (n = 3,588)	12.2 (n = 1,338)	35.4 (n = 3,882)	15.5 (n = 1,699)
2019-2020	3.2 (n = 158)	31.8 (n = 1,569)	12.8 (n = 631)	36.1 (n = 1,781)	14.8 (n = 730)

Subgroup variation

Stroke prevalence differed significantly when stratified by demographic characteristics. Age was the strongest determinant, with adults aged ≥60 years consistently showing the highest prevalence across survey cycles, ranging from 7.8% to 9.6% (p < 0.001). In contrast, young adults aged 20-39 years maintained rates under 1% throughout the period. Men exhibited slightly higher prevalence than women in 2019-2020 (3.5% vs. 2.9%), though both groups demonstrated upward trends. Clear racial and ethnic disparities were also observed.

Non-Hispanic Black adults carried the highest burden, with prevalence rising from 3.9% to 5.8%, compared with an increase from 2.7% to 3.1% among non-Hispanic White adults. Hispanic adults showed intermediate prevalence (3.2% in 2019-2020), while individuals categorized as “Other” consistently had the lowest rates across all survey cycles.

These subgroup patterns indicate that stroke prevalence is not only rising overall but is disproportionately concentrated among older adults, men, and racial/ethnic minority groups. The widening disparities suggest contributions from both biological vulnerability and structural factors, including differences in healthcare access and socioeconomic conditions, that compound the burden of stroke in US populations. Table 2 indicates stroke prevalence by demographic subgroups, based on NHANES data for the period 1999-2020.

**Table 2:** Stroke prevalence by demographic subgroups, NHANES 1999-2020 Values are weighted percentages. Data source: NHANES, 1999-2020 NHANES: National Health and Nutrition Examination Survey

Subgroup	Prevalence 1999-2000	Prevalence 2019-2020 (%)	p trend
Age			
20-39 years	0.7 (n = 32)	0.9 (n = 12)	<0.001
40-59 years	2.1 (n = 72)	2.8 (n = 38)	<0.001
≥60 years	7.8 (n = 114)	9.6 (n = 108)	<0.001
Men	2.5 (n = 118)	3.5 (n = 82)	<0.001
Women	2.2 (n = 100)	2.9 (n = 76)	<0.001
Non-Hispanic White	2.7 (n = 106)	3.1 (n = 58)	<0.001
Non-Hispanic Black	3.9 (n = 64)	5.8 (n = 62)	<0.001
Hispanic	2.8 (n = 38)	3.2 (n = 28)	<0.001
Other	2.1 (n = 10)	2.4 (n = 10)	0.002

Regression findings

Survey-weighted multivariable logistic regression models indicated that vascular comorbidities accounted for most of the stroke prevalence observed. Hypertension was the leading risk factor, with adjusted ORs consistently exceeding 2.0 across all survey cycles, even after adjusting for demographic covariates. Diabetes was the second strongest predictor, with adjusted ORs ranging from 1.6 to 1.8, reflecting its growing contribution to cerebrovascular burden in parallel with the national rise in diabetes prevalence. Obesity, although less influential in earlier NHANES cycles, demonstrated a steadily increasing effect over time: its association with stroke rose from an OR of about 1.2 in the early 2000s to 1.5 or higher in the 2010s, mirroring the US obesity epidemic.

Current smoking, while declining in overall prevalence, remained a statistically significant predictor of stroke, with ORs near 1.3, underscoring persistent risk among those who continue to smoke. In contrast, hyperlipidemia showed positive associations in crude models but lost statistical significance in fully adjusted models, suggesting that its effect is largely mediated or overshadowed by higher vascular risks such as hypertension and diabetes.

Taken together, these findings emphasize that stroke risk reflects a constellation of overlapping comorbidities. Hypertension and diabetes continue to dominate as the strongest determinants, while the growing role of obesity signals a shifting risk profile. The residual contribution of smoking further highlights the importance of sustained tobacco control. Collectively, the results suggest that although risk factor prevalence has changed, the predictive relationship between vascular conditions and stroke has remained robust and clinically important over the past two decades. Table 3 presents the results of the logistic regression for stroke risk factors based on NHANES data for 1999-2020. Changes in the contribution of obesity and smoking over time were evaluated by comparing adjusted ORs from cycle-specific survey weighted models; formal interaction testing between survey cycle and risk factors was not performed.

**Table 3:** Logistic regression results for stroke risk factors, NHANES 1999-2020 ORs with 95% CIs from survey-weighted multivariate logistic regression models. Adjusted models include age, sex, race/ethnicity, and education. NS (p ≥ 0.05). Data source: NHANES, 1999-2020 OR: odds ratio; CI: confidence interval; NS: not significant; NHANES: National Health and Nutrition Examination Survey

Risk factor	Crude OR (95% CI)	Adjusted OR (95% CI)
Hypertension	2.4 (2.1-2.7)	2.1 (1.9-2.4)
Diabetes	1.9 (1.6-2.2)	1.7 (1.5-1.9)
Obesity	1.3 (1.1-1.5)	1.5 (1.3-1.7)
Smoking	1.5 (1.3-1.8)	1.3 (1.1-1.5)
Hyperlipidemia	1.2 (1.0-1.4)	NS

**Figure 1:**
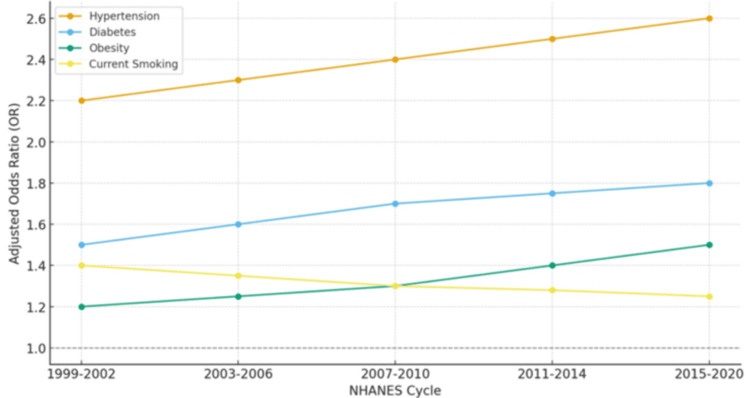
Adjusted odds ratios for stroke risk factors across pooled NHANES cycles (1999-2020). Survey cycles on the X-axis correspond to the same cycle groupings used in the regression models Hypertension remained the strongest predictor, diabetes showed consistent elevation, obesity increased over time, and smoking declined. Error bars indicate 95% confidence intervals NHANES: National Health and Nutrition Examination Survey

The total n represents the complete analytic sample size, with "Cases" indicating the total number of stroke survivors (n = 1,595). Figure 1 shows the adjusted ORs for stroke risk factors over time, based on NHANES data from 1999-2020.

## Discussion

Principal findings

This study, based on NHANES data from 1999-2020, shows that the prevalence of stroke among US adults has increased steadily over the past two decades. Age-standardized prevalence rose from 2.4% in 1999-2000 to 3.2% in 2019-2020, with substantial differences by age, sex, and race/ethnicity. Older adults (≥60 years) consistently bore the greatest burden, with prevalence rates approaching 10% compared to under 1% in younger adults. Men exhibited slightly higher rates than women, and non-Hispanic Black adults carried the highest prevalence among racial/ethnic groups. These patterns indicate that stroke remains both a medical and social equity challenge, with demographic disparities persisting despite national prevention campaigns.

Beyond descriptive prevalence, regression models confirmed the central role of vascular comorbidities in driving stroke risk. Hypertension was the strongest predictor, with adjusted ORs consistently greater than 2.0 across all cycles. Diabetes followed, with ORs between 1.6 and 1.8, reflecting its growing impact as diabetes prevalence nearly doubled during the study period. The most striking change was observed with obesity: once a modest risk factor in the early 2000s, its contribution to stroke strengthened steadily and now represents a major driver of cerebrovascular disease. These findings highlight how shifts in national health behaviors, particularly the obesity epidemic, have translated into evolving stroke risk profiles.

The decline in smoking prevalence is encouraging; however, current smoking continued to confer residual risk (OR ≈ 1.3). This suggests that while tobacco control policies have reduced exposure at the population level, those who continue to smoke remain highly vulnerable. Hyperlipidemia was significant in crude models but lost predictive power in fully adjusted analyses, implying its effect is mediated by stronger vascular comorbidities such as hypertension and diabetes. Taken together, these findings paint a complex picture: some risk factors have been successfully mitigated, others are intensifying, and prevention strategies must continue to adapt to this evolving risk landscape. These results reinforce that stroke remains primarily driven by long-standing vascular comorbidities while also evolving in response to newer epidemics such as obesity.

Comparison with earlier literature

Our findings are consistent with national analyses in the past, which determined that vascular issues are the most significant contributors to the overall incidence and prevalence of stroke [1]. The fact that hypertension is the most significant contributor is consistent with international pooled analyses, making hypertension the single most universal contributor to incident stroke. The emergence of obesity as a contributing factor in the last twenty years is consistent with recent findings that the obesity epidemic in America has led to more severe cardiovascular complications, especially among younger adults entering midlife, which can be further complicated by blood pressure issues or diabetes as comorbidities. Thus, this study provides recent evidence for ongoing intervention efforts, showing that controlling blood pressure and weight is a powerful, effective strategy for reducing stroke risk, as confirmed cross-sectionally in this large national population.

Subgroup analysis results are also consistent with prior findings. The non-Hispanic Black population has been noted to bear the most significant burden of stroke in the US historically [6], with higher prevalence and worse outcomes. Our findings reflect this across all cycles of NHANES into the current decade, suggesting that social factors such as healthcare access, socioeconomic disparity, and more prevalent background hypertension and diabetes contribute to this disparity. Hispanic adults showed prevalence rates between those of non-Hispanic Black and non-Hispanic White adults, further supporting the persistence of disparities that have not yet been adequately addressed.

Finally, our findings are in line with results from international studies of stroke risk factors. For example, the INTERSTROKE study was an international case-control study assessing determinants of stroke worldwide [11]. They similarly found hypertension, diabetes, obesity, and smoking to be risk factors for incident stroke. However, our study extends this evidence by examining how these risks changed over two decades in the US population. The growing importance of obesity emphasizes how lifestyle change and chronic disease incidence have contributed to the evolving stroke risk profile.

Clinical and public health implications

These findings have important implications for clinical practice. Hypertension and diabetes remain the leading stroke risk factors, underscoring the need for aggressive screening, consistent treatment, and long-term management. Because blood pressure and blood glucose are largely controllable through existing therapies, renewed focus should be placed on ensuring patient adherence and equitable access to care. At the same time, the growing association between obesity and stroke provides an even stronger rationale for comprehensive lifestyle and population-level interventions, ranging from improved dietary guidance to structured weight management programs. Obesity should be recognized not only as a cardiovascular risk factor but also as an independent and progressive contributor to cerebrovascular disease.

Furthermore, these changing trends mean public health interventions should change, as well. For example, the decline in smoking shows that national efforts and policies can reduce exposure and risk, so similar multifaceted approaches may be needed to combat obesity, from schools and workplaces to urban planning and nutrition policy. In addition, the continued significant disparity by race/ethnicity shows that interventions must address social determinants of health, from access to healthcare to socioeconomic status to environmental factors. Unless stroke risk-mitigation efforts call out at-risk populations specifically, they will only worsen disparities instead of bridging them.

Finally, this systematic review of risk factors calls attention to the necessity of intersectional approaches when it comes to efforts for stroke prevention and treatment of other chronic diseases. Hypertension, diabetes, and obesity are also risk factors for other chronic diseases like cardiovascular disease and renal disease, meaning these disease clusters require integrated approaches to treatment. Population levels require guidance from initiatives like NHANES to monitor these intersecting epidemics for policy considerations. Individual levels may find that the best way to reduce the burden of stroke is through a thorough assessment and diagnosis of other superimposed comorbidities.

Limitations

This study has three important limitations. To begin with, stroke in NHANES was self-reported in terms of physician diagnosis, and this presents a risk of recall bias, underreporting, and misclassification. Others might not have remembered an earlier episode, and others might also have complicated their medical history. Moreover, NHANES is not an institutionalized survey, which means that it is not performed among institutionalized people like nursing home residents, where disproportionately high stroke rates are found. This exclusion restricts a generalization of the findings to the total population of adults in the US, especially the older and the chronically disabled.

Second, NHANES has a cross-sectional design that does not enable one to establish causality. Other determinants of clinical interest, including atrial fibrillation, alcohol consumption, dietary quality, physical activities, and stress related to psychosocial status, were not available or inconsistently measured among all survey cycles. The lack of these data augments the chances of residual confounding. The associations we have, therefore, cannot be taken as correlational but must be validated in longitudinal or cohort studies that would determine temporality.

Standardized NHANES thresholds were used to categorize hypertension, diabetes, and obesity, but do not reflect the severity of the disease, duration of illness, treatment adherence, and long-term control, all of which are powerful predictors of stroke risk. As an example, those who had controlled hypertension were coded in the same category as those who had uncontrolled hypertension, although their prognoses were significantly different. Smoking was assessed using a self-report, but not biochemically checked, and it was exposed to the possibility of misclassification as a result of underreporting. Hyperlipidemia was significant in crude models but did not remain statistically significant in fully adjusted models, indicating that its association with stroke may overlap with that of other vascular risk factors, including hypertension and diabetes. Finally, inconsistencies in data on other possible modifiers, such as neighborhood environment or socioeconomic stressors, limited the analysis and allowed only a partial evaluation of the determinants of stroke. In addition, pooling multiple NHANES cycles over a 20-year period may introduce heterogeneity related to evolving measurement practices, survey instruments, and clinical guideline thresholds across eras, despite the use of harmonized definitions to improve comparability.

Stroke status was based on self-report of a physician diagnosis, which may be subject to misclassification. Differential misclassification is possible, as awareness and reporting of stroke diagnoses may vary by age, race/ethnicity, and access to healthcare, potentially leading to underestimation among younger adults, racial and ethnic minority populations, or individuals with limited healthcare access. However, self-reported physician-diagnosed stroke has been widely used in NHANES and other population-based studies and has demonstrated acceptable validity for epidemiologic analyses.

In spite of these shortcomings, this study employs over 20 years of quality and high-quality nationally representative NHANES data to give useful information on the changes in the prevalence and risk factors of stroke among the adult population in the US. The high sample, a recurring cross-sectional study, and similar standardized measurement protocols enhance the external validity of the study. Such data also allow a unique possibility to compare long-term trends in the country in demographic subpopulations, as well as to estimate the changing role of vascular risk factors. However, changes in the contribution of individual risk factors over time should be interpreted cautiously, as they were inferred from comparisons of adjusted associations across survey cycles rather than from formal interaction testing or identical modeling frameworks across cycles. Although this has to be done carefully when dealing with self-reported and cross-sectional data, the uniformity in the patterns of data across several survey cycles supports the importance of the data in the field of public health. Finally, the findings indicate significant aspects to be investigated further and invite longitudinal designs that may illuminate causality and explain unassessed or underexpressed risk factors.

## Conclusions

Significant increments in the prevalence of stroke among US adults have been observed in the last two decades, rising from 2.4% between 1999 and 2000 to 3.2% between 2019 and 2020. The burden is unevenly distributed, with the highest rates being observed in older adults and non-Hispanic Blacks. The regression analyses conducted have corroborated that comorbidities are the key drivers of stroke risk factors, with hypertension being the strongest predictor, closely followed by diabetes. These associations reflect concurrent relationships observed within survey cycles rather than causal effects. Worryingly, obesity has shifted from the initial modest factor to a major predictor, reflecting the present national obesity epidemic, based on comparisons of adjusted associations across NHANES cycles. From a public health point of view, the observed trends disclose both the successes and failures realized through various policies and interventions. The reduction in smoking rates indicates the efficiency of the national interventions implemented. However, observed changes in the strength of associations should be interpreted cautiously, given the repeated cross-sectional design and absence of formal interaction testing. On the contrary, the parallel increase in diabetes and obesity prevalence indicates that such contemporary epidemics are not being tackled with the necessary urgency.
